# Epidemiology of Hepatitis E Virus in European Countries

**DOI:** 10.3390/ijms161025711

**Published:** 2015-10-27

**Authors:** Daniele Lapa, Maria Rosaria Capobianchi, Anna Rosa Garbuglia

**Affiliations:** Laboratory of Virology, “Lazzaro Spallanzani” National Institute for Infectious Diseases, Via Portuense 292, Rome 00149, Italy; E-Mails: daniele.lapa@inmi.it (D.L.); maria.capobianchi@inmi.it (M.R.C.)

**Keywords:** hepatitis E virus, genotype, autochthonous, risk group

## Abstract

Over the last decade the seroprevalence of immunoglobulin (IgG) anti hepatitis E virus (HEV) has been increasing in European countries and shows significant variability among different geographical areas. In this review, we describe the serological data concerning the general population and risk groups in different European countries. Anti-HEV antibody prevalence ranged from 1.3% (blood donors in Italy) to 52% (blood donors in France). Various studies performed on risk groups in Denmark, Moldova and Sweden revealed that swine farmers have a high seroprevalence of HEV IgG (range 13%–51.1%), confirming that pigs represent an important risk factor in HEV infection in humans. Subtypes 3e,f are the main genotypes detected in the European population. Sporadic cases of autochthonous genotype 4 have been described in Spain, France, and Italy. Although most HEV infections are subclinical, in immune-suppressed and transplant patients they could provoke chronic infection. Fulminant hepatitis has rarely been observed and it was related to genotype 3. Interferon and ribavirin treatment was seen to represent the most promising therapy.

## 1. Introduction

HEV (hepatitis E virus) represents the main cause of enterically transmitted hepatitis worldwide, being responsible for more than 50% of the cases of acute hepatitis in endemic countries. For example, it is responsible for more than 50% of the acute hepatitis infections in India, about 25% in Africa, and 15%–20% in Eastern-Oriental countries [1]. A study estimated that the HEV global infection burden is 3.4 million symptomatic cases of hepatitis E each year, with 70,000 deaths and 3000 stillbirths [2].

HEV is principally transmitted through the fecal-oral route because of fecal contamination of drinking water. Other transmission routes have been identified, and include: food-borne transmission by ingestion of meat from infected animals; transfusion of infected blood products, and vertical transmission from pregnant women to their fetuses. In developing countries, contaminated water causes serious epidemic outbreaks [3]. For this reason, hepatitis E was considered to be one of the many diseases linked to the poverty of tropical and subtropical countries [4].

In Europe, there is much evidence indicating that HEV is prevalently transmitted by the ingestion of pork and wild boar meat, but other sources of infection such as blood transfusion cannot be excluded [5].

In this review, we would like to provide an overview of the diffusion of human HEV in Europe. In the first part of the review, we describe the general aspects of HEV virus, serological data in European countries, and HEV genotypes circulating among humans, while in second part we describe HEV chronic infection in immune-depressed patients and the therapies used in their care.

## 2. General Aspects of HEV (Hepatitis E Virus)

HEV was discovered using immune electron microscopy in 1983 by Balayan [6]. Balayan was charged to investigate a non-A, non-B hepatitis outbreak which occurred in a Soviet army camp located in Afghanistan. To assess if the causative agent of this outbreak could be transmitted from human to human, Balayan himself ingested pooled stool from extracts of nine patients with Afghan outbreak hepatitis. He developed typical symptoms of acute hepatitis 36 days after stool extract ingestion. Spherical viral particles measuring 27–30 nm were identified in stool samples drawn on days 28, 43, 44 and 45 after inoculation. This was the first description of virion. Shortly thereafter, this virus was identified in serum derived from patients involved in a large epidemic of water-borne hepatitis in New Delhi (India) between 1955 and 1956, initially defined as non-A, non-B epidemic hepatitis [7]. Subsequently, even the stored sera from an outbreak occurring in Kashmir from 1978 to 1979 resulted HEV positive. The virus was named E virus because of its enteric route of transmission and its ability to cause epidemics.

HEV is a single-stranded, positive-sense RNA virus of approximately 7.2 kb in size and capped. It belongs to the genus *Hepevirus*, the only member of the family *Hepeviridae* [8]. Its capsid is icosahedral and it does not possess an envelope.

Two main species of the virus are recognized: (1) mammalian HEV; it includes genotypes 1 and 2 that infect only human beings, and genotypes 3 and 4 which could have many mammalian species as a reservoir other than humans, especially pigs, wild boars, deer, rabbit as well as other mammals (rabbit, cow, bat); (2) avian HEV (genotype 5), which causes severe liver and spleen disease in chickens; it is well known to infect other birds such as the turkey, and it has a low genetic identity with other genotypes (<50%) [9].

The HEV viral genome includes two short non-coding regions at 5′ and 3′ untraslated regions (UTR)with a poly(A) tail [10]. They contain *cis*-acting elements which have important roles in HEV replication and transcription [11].

The coding region consists of three discontinuous and partially open reading frames (ORF1-3).

The genome is well characterized in the Burmese strain (genotype 1). ORF-1 encodes non-structural proteins involved in replication and processing, including an RNA helicase, an RNA-dependent RNA polymerase, a methiltransferase, and a cystein protease [12]. It extends approximately 5 kb from the 5′ end. ORF-2 consists of approximately 1980 nt and encodes for capsid protein. It occupies the 3′ end of the coding region. ORF-3 is the smallest ORF, with a maximal coding capacity of 372 nt; it overlaps ORF1 by one nucleotide at the 5′ end and overlaps ORF2 by 331 nt at the 3′ end. ORF3 encodes a small immunogenic protein with a maximum length of 123 amino acids. The ORF3 functions are uncertain. It has been claimed that the ORF3 protein is not necessary for replication and virus assembly while it probably does have a role in cellular environment regulation [13].

HEV genotype 1 is the cause of severe clinical fulminant hepatic failure in pregnant women and is associated with high mortality, particularly in the third trimester, and in obstetric complications such as eclampsia or hemorrhage [12,14].

This genotype is endemic in Africa and Asia, but it is frequently isolated from hepatitis E cases among travelers to these regions from non-endemic areas. The strains belonging to this genotype have a >90% sequence homology. Five subtypes 1a–e have been recognized, with different prevalences in different geographical areas. Genotype 1a is the most frequent subtype1. It is diffused in many Asian countries (Nepal, Pakistan, Bangladesh, Vietnam and India). Subtype 1b has been predominantly identified in China, and it is frequently found in China, Pakistan [15], and Bangladesh [16], but also in Caribbean countries such as Haiti [17] and Cuba [18]. Subtype 1c is prevalently detected in China [19] and India [20], but some cases are also described in Kyrgyzstan [21]. Subtypes 1d,e, have been exclusively described in Africa [22,23]. Only one patient in Spain with HEV infection harbored genotype 1e indicating that this case represented an imported infection given that the patient had visited Ethiopia a few weeks before appearance of symptoms [24].

The strains belonging to genotype 2 are exclusively human strains. We know two subtypes: subtype 2a, isolated in Mexico and considered to be the prototype strain [25], and subtype 2b which has been observed in several African countries, mainly Nigeria and Chad [26]. The genotype 2 isolates have a nucleotide homology of only 75% with genotype 1 isolates [23,27].

Genotype 3 was first identified in human cases of locally acquired hepatitis E in the USA [28,29]. Genotype 3 is diffused worldwide. In Europe it represents the most frequently detected genotype.

Subtype 3a has been described in the United States [30], Japan [31], Korea [32], and Holland [33]. Genotype 3b has been isolated in Japan, China, and Canada while subtype 3c is mainly described in France [34,35], Germany [36], Holland [33], and Italy [37]. Genotype 3d is detected in Taiwan and the United States [38]. Subtype 3e is considered to be an autochthonous European strain. It is frequently described in the United Kingdom (UK) [39] Holland [33], Italy [40,41], Greece [42], and Spain [43]. In addition, strains from wild boar were isolated in Japan and Thailand [44,45]. Genotype 3f has been described in most of the European countries (Spain, France, United Kingdom, Greece, the Netherlands, Italy) [37], but also in Japan [27], Thailand [46], and New Caledonia [47]. In Kyrgyzstan 3g subtypes have been isolated [48]. Subtype 3h has been described in Italy [49,50], Uruguay [51], and New Zealand [52]. Subtype 3i has been described in Latin America [51,53,54] but also in Europe: Austria [55], Italy [41], and France [56]. Subtype 3j has been described on different continents in: Canada [57], Australia, and Mexico [58].

Genotype 4 is prevalently diffused in China, Taiwan, Vietnam, and Japan, where we could find subtypes 4a–d. However, subtype 4b had been described in Belgium (pig isolate), and subtype 4d is mainly diffused in China, but also found in Italy, in both humans and swine [59,60]. Subtype 4c has been described in India, 4f in Japan and Vietnam and an imported case has been described in Germany [61]. Subtype 4g has only been described in China [62]. Strains of genotype 3 and 4 have been isolated in several animals such as pigs, cows, wild boars, and deer, all representing the main reservoirs of 3–4 HEV genotypes.

## 3. Clinical Features

Illness caused by HEV is difficult to discern from hepatitis A virus (HAV) to but patients with severe HEV related symptoms tend to be older than those observed in hepatitis A cases [63]. Acute HEV infection was defined as a resolution within the first six months after diagnosis. Chronic HEV infection was defined as the persistence of HEV-RNA detection for longer than six months which could lead to cirrhosis [64]. The initial symptoms of acute hepatitis E are typically unspecific and include: flu-like myalgia, arthralgia, weakness, and emesis. Some patients present fever, vomiting, nausea, anorexia, abdominal pain, and jaundice accompanied by elevated bilirubin and liver transaminase, alkaline phosphatase and glutamyltransferase activities.

In most instances, acute HEV 3 and HEV 4 are self-limiting illnesses and the patient recovers without sequel; though case fatality rate in the general population varies between 0.1% and 3% [12]. Symptomatic infection may be misdiagnosed. For example, HEV can be mistaken for drug-induced liver injury. A study in the UK showed that 6 (13%) out of 47 patients with “criterion-referenced” drug-induced liver injury had been incorrectly diagnosed, and in fact, the patients had acute HEV genotype 3 infection [65]. Furthermore, most HEV infections are asymptomatic, considering its high seroprevalence in some developed countries, such as the UK, Germany, and France, and the relative rarity of clinically diagnosed infections. This is supported by data from an outbreak on a cruise ship, where 67% of patients were asymptomatic [66].

As with other viral hepatitis, extra-hepatic manifestations can occur and the spectrum of clinical disorders is still emerging. In fact, neurological disorders have been found to be associated with locally acquired acute and chronic HEV infection in the UK and France [67,68]. Among the 126 patients with acute HEV hepatitis observed in hospital from 2004 to 2009, 5.5% developed neurological complications [67]. Furthermore, Guillain-Barrè syndrome is frequently reported. Tse *et al.* described a case report with Guillan-Barrè syndrome associated with acute hepatitis E infection in China [69].

Chronic infections are observed among transplant patients. Many factors are associated with failure of immune-suppressed transplant recipients to clear HEV after acute infection factors include the degree of immune-suppression, the time between the last episode of acute rejection and HEV infection, time since transplantation, low leukocyte level, and low total-lymphocyte count [70].

## 4. Seroprevalence in the General Population and Risk Groups for HEV Infection

In Europe, acute HEV infection is diagnosed in 5%–15% of patients with acute hepatitis for whom hepatitis A–C had been ruled out [65,71,72]. Most patients with acute hepatitis E virus are travelers in endemic areas, or those who have undergone transfusions; they also commonly consume pig meat or drink contaminated water. Over the last decade, the seroprevalence of anti-HEV IgG has been increasing in European countries and shows significant variability among different geographical areas. The main studies have been carried out on the general population and on blood donors, and the high prevalence of HEV-RNA positive infection and anti-HEV antibody prevalence in blood donors between 1% and 52% suggests that HEV is responsible for several cases of subclinical HEV infection in Europe [73,74,75,76,77].

In a seroprevalence study carried out by Norder, antibodies against hepatitis E were found in 248 Swedish and Danish subjects between 1993 and 2007, 163 only harbored IgG (109/163 were males) and 85 tested positive for immunoglobulin M (IgM) and IgG together (57/85 were males); the mean ages were 43.5 and 31.5 years, respectively. HEV-RNA was found in 65 patients [78]. A second seroprevalence study, which was carried out in Sweden, revealed that HEV IgG prevalence was 13% (15/115) and 9.3% (10/108) in pig farmers and control subjects, respectively [79].

In Finland, in a study carried out on 97 patients with diagnosed acute unexplained non-A, non-C hepatitis between 2000 and 2008, 27.6% of all serum samples were positive for anti-HEV antibodies: 11/97 (11.3%) were found positive for anti-HEV IgG only, suggesting a past infection, but 11 of them (11.3%) were HEV-RNA and/or IgM positive [80].

In Southwest England, a study carried out from 2000 to 2006 included 500 blood donors to trace the evolution of the anti-HEV antibody prevalence. The anti-HEV IgG prevalence was 16% [81]. Recently, anti-HEV IgG and IgM seroprevalence were also determined in 1559 Scottish blood donors: 4.7% (73/1559) were found IgG positive, none tested positive for IgM [82]. In Denmark, different anti-HEV IgG seroprevalences were found among farmers, who worked with pigs 144/286 (50.4%), and blood donors, 94/456 (20.6%) [83]. A study carried out in a population of Danish prisoners and drug users showed an anti-HEV IgG seroprevalence of 16.9% using an in-house assay. The assay used to test anti-HEV IgG was based on ORF2 of the Pakistani HEV strain SAR-55 expressed in baculovirus. The seroprevalence dropped to 4.1% with the Abbott commercial assay [84]. In 2011, in Belgium, the anti-HEV IgG seroprevalence was 14% from a total of 100 patients attending the gynecological or orthopedic clinics [85], instead in Russia, a 18.2% anti-HEV IgG seroprevalence was observed in 62/341 children [86].

In Germany, several anti-HEV seroprevalence data have been reported. A study carried out from 2008 to 2011 analyzing the prevalence in an adult population of subjects ranging in age from 18 to 79 years showed an anti-HEV IgG positivity of 16.8%: the prevalence increased with age, leveling off at >60 years of age [87]. In another study, the anti-HEV IgG seroprevalence was 6.8% (69/1019) in blood donors [88]. Between 2006 and 2007, 76/96 people with HEV infection were reported in a routine surveillance system. Most people with travel-associated infection and autochthonous infection described in this study were males (62% and 76%, respectively). Individuals with travel-associated diseases were younger (median age, 37 years) than those with autochthonous infections (median age, 46 years). Genotypes 3 or 4 were present in 15/15 subjects with autochthonous infection, and genotype 1 in 8/9 subjects with travel associated infections [61].

In 2008, in Eastern Germany, in a study on anti-HEV IgG, a seroprevalence of 11% and 18% was found among blood donors and forestry workers, respectively [89].

In Poland, sera from 182 patients were tested for anti-HEV IgG and IgM. IgG positivity was found in 15.9% (29/182) of them, and three sera were reactive for HEV IgM only [76].

In Moldova, a study on seroprevalence was conducted from 1997 to 1998 in two populations: swine farmers (*n* = 264) and a group of people without occupational exposure to swine (*n* = 255), who were considered as the control group. There was a higher prevalence of anti-HEV IgG in the swine farmer group compared with the control group (51.1% *vs.* 24.7% *p* < 0.001). In the occupational exposure group, people >40 years had a significantly higher prevalence of HEV infection than those aged 18–30 years (65.7% *vs.* 40.0% *p* = 0.006). A similar pattern of association between anti-HEV reactivity and age was noticed in the control group (33% *vs.* 17.9%) [90].

In the years from 1993 to 2005, 12,091 cases of communicable disease were imported to the Czech Republic and 5% were related to HEV [91]. In another study carried out among patients with acute hepatitis symptoms and who resulted negative for antibodies against A–C hepatitis, 27.8% of them resulted positive for anti-HEV IgG [92].

In the Netherlands, anti-HEV IgG seroprevalence resulted to be 11% for pig veterinarians, 6% for non-pig veterinarians and 2% in the general population, indicating that direct exposure to swine may be an important risk factor for HEV infection [93]. Again, in this country, 5239 blood donors were tested for the presence of anti-HEV IgG and IgM in four districts (north-east, south-east, north-west, and south-west) of the Netherlands between 2011 and 2012. HEV-RNA was tested in anti-HEV IgM positive samples. In total, 26.7%, (1401/5239) of the blood donors were reactive for anti-HEV IgG; moreover, 3.5% (49/1401) also resulted positive for anti-HEV IgM and only 4/49 IgM positive were HEV-RNA positive. Furthermore it was observed that the seroprevalence in the south-eastern geographic area was higher (30.5%) than in the rest of the country (*p* = 0.0004), while in the north-western part it was lower (23.6%, *p* = 0.009). Anti-HEV IgG seroprevalence strongly increased with age after the age of 30 increasing by 1.05% per year. The overall seroprevalence in males was higher than in females (29.2 *vs.* 23.1%), but this difference could be attributed to the higher age of males donors (51.1 *vs.* 45.5 years) [94].

In France, many studies were carried out in different geographical areas. In Southwest France, different populations were studied. Anti-HEV IgG seroprevalence carried out among 529 blood donors from Midi Pyrenée revealed 16.6% IgG positivity and none of the subjects had traveled abroad in the previous six months [95]. In the Pyrenees region, in 2001–2002, among 431 patients with acute hepatitis of unknown etiology, 10.7% (46/431) resulted to be anti-HEV IgG positive while none of them had travelled to endemic countries. Phylogenetic analysis demonstrated that all the strains belonged to genotype 3 [96].

In Alsace-Lorraine, Franche-Comtè, Champagne-Ardenne, and Bourgogne administrative regions, anti-HEV IgG were detected in 31.2% of the forestry workers from 2002 to 2003 [97], while in the Toulouse region the seroprevalence in blood donors rose to 52% when a more sensitive assay was used [77]. In another study carried out in the southwest of France in the Midi-Pyrenees region, the seroprevalence anti-HEV IgG from blood donor sera was 3.2% [98].

Anti-HEV antibodies were determined among Austrian adults, including 407 professional soldiers and 590 civilians, (age range, 18–59 years, 980 males and 17 females).The overall sero-positivity for HEV antibodies was 14.3% and increased with age. In fact, among individuals aged up to 19 years, the seroprevalence was 8.1% and increased to a seroprevalence rate of 57.5% among individuals aged 50–60 years [99], while in Southwest Switzerland in 2011, the anti-HEV IgG prevalence among 550 blood donors was 4.9% (27/550) [100].

In Romania, the anti-HEV IgG prevalence was 12.5% and 14% in students and health workers (doctors and nurses), respectively [101].

In southeast Hungary, between 2001 and 2006, 9.6% patients with an average age of 53 years (age range: 16–85 years), showing acute hepatitis symptoms, were positive for anti-HEV IgM (116/1203), and 24.5% of them were also HEV-RNA positive [102].

In Spain, the HEV seroprevalence was determined in 863 voluntary Spanish blood donors (age range: 15–65 years; 56% males). A total of 34/863 serum samples (3.9%) resulted positive to anti-HEV IgG: 25 of these were confirmed by Western blot. Anti-HEV IgM was not present in any of the anti-HEV IgG positive serum samples [103]. A lower IgG seroprevalence (1.08%) was reported in another Spanish study performed on 2305 serum samples collected in 2008 from the general population (age range: 2–60 years) [75].

Again in Spain, 277 subjects with acute non-A- non-C hepatitis of unknown etiology were analyzed for anti-HEV antibodies. Evidence of acute infection by HEV was obtained for a total of 30 patients (10%), and 16 cases were unrelated to recent international travel. On samples from among 158 patients tested for both anti-HEV IgM and HEV-RNA at admission, the average of IgM antibody was 11.4%, while only 9.5% of the samples resulted HEV-RNA positive [104].

In 1998 in Portugal, Duque *et al.* carried out the first serological study to assess the presence of anti-HEV IgG in 50 blood donors and the results showed that 4% (2/50) were anti-HEV IgG positive [105]. In this country, the first cases of acute autochthonous hepatitis E virus in humans were described in 2010 [106].

The data on the prevalence of HEV infection and the prevalence of circulating HEV antibodies in Italy are limited. In Northern Italy, in Milan, between 1994 and 2009, 651 patients with acute non-A- non-C hepatitis were analyzed; 20.6% (134/651) of the patients were HEV positive. All of them were anti-HEV IgM and IgG positive and 71.6% (96/134) were also positive for HEV-RNA. Furthermore, 6% (39/651) were anti-HEV IgG positive, but negative for anti-HEV IgM. A total of 81.3% of the patients (109/134) showed hepatitis E while travelling to endemic areas, 2.3% (3/134) acquired intra-familial infection from relatives who developed travel-related diseases, whereas 16.4% (22/134) patients affirmed no having travelled abroad [107].

In Southern Italy, HEV seroprevalence was analyzed in a group of 805 Italian people divided into four groups: 151 volunteer blood donors (mean age: 37.0) with an anti-HEV IgG seroprevalence of 1.3%, 450 subjects from the general population (mean age: 40.1) with an anti-HEV IgG seroprevalence of 2.7%, 100 HIV-positive with an HEV IgG seroprevalence of 2.0% (mean age: 39.1) and 104 haemodialysis patients (mean age: 65.1) with an HEV IgG seroprevalence of 9.6%. The mean anti-HEV IgG seroprevalence was 3.9% [108]. In the city of Lecce (Southern Italy), a general population group showed an anti-HEV IgG seroprevalence of 2.9%, while in drug users and hemodialyzed patients the seroprevalence rates were 0.7% and 4.3%, respectively [109].

In another study carried out in Italy between 1994 and 1997, HEV-RNA and anti-HEV antibodies were analyzed in 218 subjects (123 males and 95 females with a mean age of 37.5 years) diagnosed with acute viral non-A, non-C hepatitis. The results showed that 10.1% (22/218) of the examined patients were diagnosed with acute hepatitis E; the main risk factor was traveling in an endemic area. In fact, 77.3% (17/22) of the HEV positive people developed acute hepatitis after their return from endemic areas (eight patients from India, three patients from Pakistan, five patients from Bangladesh, and one patient from Somalia). One five year-old child developed acute hepatitis E two days after his return from Somalia. Four patients (18.2%) denied traveling abroad, and excluded having had contact with people coming from endemic areas or having been exposed to other risk factors such as drug addiction or shellfish consumption [40].

In another study performed in Southern Italy, 6% of the cryptogenic acute hepatitis cases were associated with HEV infection [110]. A recent Italian study on anti-HEV seroprevalence, which was carried out among people at risk of HIV infection, showed an anti-HEV IgG seroprevalence of 5.38%. The anti-HEV IgG positivity was not correlated with HIV infection [111].

In Serbia during the spring of 2010, serum samples were collected from 200 volunteer blood donors ranging from 19 to 65 years of age (average age: 39.3). A total of 30/200 (15%) of the blood donors tested positive for anti-HEV IgG. No significant differences of anti-HEV IgG seropositivity were found between men and women (14.6% and 16.7%, respectively) [112].

In Albania, a case-study was carried out to evaluate the seroprevalence of anti-HEV antibodies involving 109 patients with chronic liver disease, and 190 patients with no liver disease manifestation to be considered as a control group. The anti-HEV IgG seroprevalence was 36.6% among people with chronic liver disease (40/109) and 12.1% among controls (23/190). None of the anti-HEV IgG positive patients had traveled to areas endemic for HEV infection [113].

In a population in Northwestern Greece (Epirus region) the seroprevalence of anti-HEV IgG estimated was 4.85% among refugees from Southern Albania, 5.3% in HBV chronic patients and 1.34% in hemodialysis patients [114].

Many studies have been performed in Turkey on HEV seroprevalence. Two studies analyzed seroprevalence in pregnant women. The first paper included 245 pregnant women between 17 and 41 years of age, and 76 healthy women between 19 and 42 years of age, used as the control. Antibodies IgG were found in 12.6% of the pregnant women (31/245), and 11.8% (9/76) in the control group [115]. In a second study, serum samples from 386 pregnant women were screened for anti-HEV antibodies. IgG was found in 7.0% (27/386) of the samples [116]. Two other Turkish studies were conducted on children. First, 515 students in a primary school in Ankara, with a median age of 7 years, were examined at two different times, once in November, 2003 and once in January, 2005. The seroprevalence of anti-HEV IgG was 1.7% and 2.1% at the first and second visits, respectively [117]. In the second study, 210 healthy children and young people were randomly selected in Konya (Anatolia region) with an age range of 1–18 years (100 lived in rural areas, where socio-economic conditions are poor and the purification system is known to be unreliable, and 110 lived in urban areas. The HEV antibody IgG was 8.5% in rural areas and 5.2% in urban areas [118].

Another study suggested that a particular risk group for hepatitis E infection could be represented by agricultural workers, who use contaminated water for irrigation [119]. In this study, two groups of people were considered: 46 farm workers and 45 control subjects. The median age was 27.6 years in the farm workers group and 28.5 years in the control group, most of them were males. None of the people had a recent history of jaundice or clinical illness. No information was given on how long the subjects had been exposed to waste water. The anti-HEV IgG seroprevalence was 16/46 (34.8%) in the agricultural farm workers, and 2/45 (4.4%) in the control subjects. The risk of acquiring hepatitis E was 11.5 fold higher in farm workers than in the controls [119].

The main data concerning HEV seroprevalence are represented in Table 1.

**Table 1:** Hepatitis E seroprevalence in general populations and risk groups in European countries.

Country	Population	Seroprevalence (%)	Assay	Reference
Sweden	BD	9.3	Abbott HEV EIA	[79]
	*SW*	13.0		
Finland	*PT*	11.3	Genelabs	[80]
England	BD	16–25	Genelabs	[81]
Scotland	BD	4.7	Wantai	[82]
Denmark	BD	20.6	In house assay	[83]
	P	16.9	In house assay	[84]
	*D*	4.1	Abbott HEV EIA	[84]
	*SW*	50.4	In house assay	[83]
Belgium	*PT*	14.0	Biorex diagnostics	[85]
Russia	CH	18.2	In house assay	[86]
Germany	AD	16.8	Mikrogen	[87]
	BD	6.8	Mikrogen	[88]
	BD	11.0	Mikrogen	[89]
	*FW*	18.0	Mikrogen	[89]
Poland	*PT*	15.9	Adaltis	[76]
Moldova	*SW*	51.1	In house assay	[90]
Czech Republic	*AH*	5.0	Not done	[91]
	*AH*	27.8	Abbott HEV EIA	[92]
The Netherlands	BD	26.7	Wantai	[94]
	*PV*	11.0	Abbott HEV EIA	[93]
	*NPV*	6.0	Abbott HEV EIA	[93]
France	BD	52.0	Wantai	[77]
	BD	16.6	Genelabs	[95]
	BD	3.2	Genelabs	[98]
	*AH*	10.7	Abbott HEV EIA	[96]
	*FW*	31.2	MP Biomedicals	[97]
Austria	C-S	14.3	Wantai	[99]
Switzerland	BD	4.9	Genelabs	[100]
Romania	ST	12.5	Mikrogen	[101]
	HW	14.0	Mikrogen	[101]
Hungary	*AH*	9.6	HEV Ab, Dia.Pro	[102]
Spain	BD	1.08	HEV Ab, Dia.Pro	[75]
	BD	3.9	Abbott HEV EIA	[103]
	I	5.5	Abbott HEV EIA	[103]
	*PT*	11.4	Mikrogen	[104]
Portugal	BD	4	Abbott HEV EIA	[105]
Italy	I	3.9	HEV Ab Dia.Pro	[108]
	BD	1.3	HEV Ab Dia.Pro	[108]
	*D*	0.7	Abbott HEV EIA	[109]
	*AH*	10.1	Abbott HEV EIA	[40]
	*AH*	20.6	Genelabs	[107]
	*AH*	6.0	HEV Ab Dia.Pro	[110]
	AD	5.38	HEV Ab Dia.Pro	[111]
Serbia	BD	15.0	In house assay	[112]
Albania	*CLD*	36.6	Abbott HEV EIA	[113]
Greece	R	4.85	Abbott HEV EIA	[114]
	*CVHB*	5.3		
Turkey	PW	12.6	Virotech	[115]
	PW	7.0	Globe Diagnostics	[116]
	CH	2.1	HEV Ab Dia.Pro	[117]
	CH	8.5	Not done	[118]
	*AW*	34.8	Biyoser S.r.l.	[119]

General populations: BD, blood donors; P, prisoners; CH, children; AD, adult population; ST, students; HW, health workers; C-S, civilians and soldiers; I, Italians; R, refugees; PW, pregnant women; Risk groups (in italics): *SW*, swine workers; *D*, drug users; *FW*, forestry workers; *PT*, patients; *AH*, acute hepatitis; *PV*, pig veterinarians; *NPV*, non-pig veterinarians; *CVHB*, patients with chronic viral hepatitis B; *CLD*, chronic liver disease; *AW*, agricultural workers.

## 5. Anti-HEV Antibody Seroprevalence and Chronic HEV Infection Features in Immunocompromised Patients

HEV is generally self-limiting, except in immunosuppressed people. Immunocompromised patients are at particular risk of fulminant hepatic failure upon HEV infection, and chronicization of HEV infection has been observed. In Europe, chronic hepatitis E cases have been described in HIV infected patients [120,121,122], solid-organ transplant recipients (SOT) [64,122], patients with hematological disease and patients with hematological disorders who had undergone chemotherapy [123]. Sometimes, chronic infection may also lead to cirrhosis [64,124].

Different findings have been reported on the prevalence of anti-HEV IgG antibody in hemodialysis patients living in developed countries [125,126,127,128].

In an English study, anti-HEV IgG/M and HEV-RNA positivity was investigated in 76 hemodialyzed patients (45 males and 31 females) with a median age of 70 years, and in 88 renal transplants (55 males and 33 females) with a median age of 55 years; they were compared with 670 in the control group, which included healthy people, >18 years old with no history of renal or liver dysfunctions. The control group was only investigated for anti-HEV IgG. Anti-HEV IgG was positive in 36.8% (28/76) of the hemodialysis patients and in 18.2% (16/88) of the transplant patients. HEV-RNA was not detected in any of the patients. In the control subjects, 18.8% (126/670) of the subjects were anti-HEV IgG positive [129].

In an anti-HEV seroprevalence study in the UK, 138 HIV positive patients were compared with 464 patients, aged ≥18 years, to be considered as control subjects. These patients had no history of liver disease [130]. Among the 138 HIV positive, 79% patients (109/138) were males with a median age of 43 years. Thirteen out of 138 were anti-HEV IgG positive (9.4%), compared with 13.8% among the control patients (64/464). Anti-HEV IgG in the control group showed an increased seroprevalence in older people. Notably, in this study there was no difference in anti-HEV IgG seroprevalence between the HIV infected patient population and the control group [130].

In the Netherlands, an anti-HEV IgG of 11.7% was observed among 231 HIV infected patients [131].

In Germany, a study showed an anti-HEV IgG seroprevalence of 2.4% in pediatric renal transplant recipients, and 4.9% in pediatric liver transplant recipients [132].

In a study carried out in Switzerland, 2.6% of the 735 HIV patients were reported to be positive for anti-HEV antibodies [133].

In a French study on SOT patients, the anti-HEV IgG seroprevalence was 14.4% for kidney recipients and 10.4% for liver recipients [134]. Similar averages were reported in a study done in Southeastern France, where an anti-HEV IgG seroprevalence of 6% was observed [135]. Another study described the outcomes of HEV infections in a cohort of 1350 kidney transplant recipients living in Southeastern France between 2007 and 2011. The median patient age was 51 years. Sixteen HEV infections were diagnosed with genotype 3c,e,f, and moderate transaminases or an increased glutamyl transferase (GT) level was observed in all of the cases. All patients were infected with autochthonous strains and one patient developed liver cirrhosis 14 months after infection [136]. Generally, in organ transplant patients chronic HEV infection shows a rapid progression towards liver fibrosis in 10% of the cases [70].

A study investigated the seroprevalence of anti-HEV antibodies in two distinct immune-compromised patient populations: (1) 261 samples belonged to HIV infected subjects (82% were males) with a median age of 41 years; and (2) 46 samples were collected from kidney transplant patients (66% were males) with median age of 45 years. Anti-HEV IgG positivity was found in 1.5% of the HIV infected patients (4/261) and 6.5% (3/46) of the kidney transplant patients. Anti-HEV IgM antibodies were all negative, therefore excluding any acute infection; the IgG avidity index confirmed previous HEV infection among the tested patients [137].

A retrospective study was carried out in 184 HIV-positive patients who were followed-up at the University hospital of Marseille in Southeastern France [138]. Among these patients, 119 were men; their mean age was 42 years. The prevalence of anti-HEV IgG and IgM was 4.4% (8/184) and 1.6% (3/184), respectively [138].

Another study on anti-HEV IgG seroprevalence was carried out on a total of 245 HIV infected patients, 133 living Southern France and 112 in Northern France. A higher seroprevalence was observed in the southern patients (9%) than in the northern patients (3%) [139].

In Italy an anti-HEV seroprevalence of 5.7% was described in HIV positive people [140].

In a study carried out in Spain on 238 selected HIV positive cases, 9% (22/238) of them were anti-HEV IgG positive, but none resulted IgM positive [141]. Mateos-Lindemann observed an anti-HEV IgG positivity of 10.4% among the 448 HIV infected patients, whereas only one patient resulted as HEV-RNA positive [142].

In another more recent Spanish study, the anti-HEV seroprevalence in HIV positive patients was 9.6% [143].

In 2001, in a semi-rural geographic area of Greece (Thessaly region), the anti-HEV antibody prevalence among 351 hemodialysis patients (264 males with a mean age of 60 ± 14 years) resulted 4.8% (17/351); no association was found between positivity, age, and sex [127]. Instead, another seroprevalence study carried out on 243 HIV infected patients showed an anti-HEV seroprevalence of 7.3% [144].

## 6. HEV Genotype Distribution among Reported Cases of HEV Infection in Humans

Autochthonous hepatitis E infection is an emerging disease in Europe and is generally caused by genotype 3, while genotype 4 has rarely been described [61,84,145,146,147,148]. Severe cases of fulminant autochthonous hepatitis E caused by genotype 3 have been reported; they often occurred in immune-compromised subjects or in those with chronic liver disease (Table 2) [149,150].

Several case reports on HEV imported cases have been described in many countries: in the UK from India and Saudi Arabia [151,152], in the Netherlands from Bangladesh, Somalia, and the Middle East [153], and Sweden and Turkey [154]. These reports published in the 1990s described single patient cases and clinical features similar to those in endemic countries. All HEV strains linked to these imported infections were genotype 1.

Other studies were conducted in the UK. In England and Wales, between 1996 and 2003, 478 subjects were tested for anti-HEV IgM/IgG. A total of 186 hepatitis E cases were serologically diagnosed; and 9% (17/186) of them were not associated with recent travel abroad. A total of 69% (129/186) of the cases were associated with travel to countries where HEV is hyper endemic. The non-travel associated cases were infected by HEV genotype 3 strains [155].

In a Dutch study, which was carried out among patients with HEV-RNA positivity, viral genotyping revealed that all isolates belonged to genotype 3, mostly being genotype 3c, whereas genotypes 3f, 3a, and 3e were less frequently detected [156].

Another study included 333 patients with non-A-non-C hepatitis who were tested for HEV infection markers over a seven-year period; their median age was 67 years (range 35–83). Autochthonous hepatitis E genotype 3 strains were identified and they were close related to the HEV strains circulating in English pigs [157].

Between 2006 and 2012, several sporadic cases of autochthonous HEV-infection were reported in different areas in Germany and viral genotyping revealed that all sequences corresponded to HEV genotype 3 [158,159,160,161].

Between April and August 2010, a total of 200 raw porcine liver samples were purchased in 81 butcher shops and grocery stores in Regensburg (Southeastern Germany). Specimens from 4% (8/200) had detectable amount of HEV-RNA. Phylogenetic analysis indicated the presence of two novel isolates of HEV genotype 3, subtype a (swR437) and c (swR269), respectively. Both novel swine HEV isolates showed high sequence homology to isolates from patients with acute HEV infection from the same geographic region. These results support the role of undercooked pig products as a source of zoonotic HEV infection in humans [36]. Recently, an autochthonous HEV 3c subtype infection was reported in a 27 year old German pregnant woman; this was closely related to other European isolates [162]. In a German study carried out on 16,125 blood donors, 0.08% of samples were found to be HEV-RNA positive (13/16,125) and all donor sequences resulted to be genotype 3a, 3c or 3e [163].

In the Czech Republic, genotypes 3e, 3f and 3g were identified from 10 human clinical samples. Their genetic relatedness with Czech animal strains suggests an autochthonous source, likely linked to the consumption of contaminated pork [164]. Recently, 51 adult patients with HEV were evaluated in a study carried out on Czech Republic hepatitis between the years 2009 and 2012. Anti-HEV immunoglobulin was identified in 98% of the patients (50/51). Genotype 3 subtypes a, c, e–g were frequently observed. These human strains were closely related to local swine and wild boar strains [165].

In France, subtypes of genotype 3 have often been isolated in humans.

The first human case of 3c hepatitis E infection was reported in a 46 year old man, who presented a two day history of diffuse polymyalgia and arthralgia. A neurological examination was normal. Biological tests revealed increased levels of liver enzymes. The patient did not report any recent travel in HEV hyper endemic areas [166].

In Marseille, HEV infection were confirmed by anti-HEV IgM testing and detection of HEV-RNA in serum samples of 11 patients with acute hepatitis. The mean age of the patients was 57 years. Of the 11 case-patients, 10 were males and 3 were also kidney transplant recipients. HEV infection was clinically asymptomatic in all transplant recipients; the infection was diagnosed after routine transplant laboratory tests. Phylogenetic analysis showed that the four patients had either HEV genotype 3c or 3f [146]; instead, HEV genotype 4 RNA was found in two patients who had eaten uncooked pork liver sausage [146].

A 77-year-old woman presented the first case of fatal fulminant liver failure associated with HEV infection, however the woman suffered of autoimmune hepatitis and she ingested excessive paracetamol drug. The patient did not report any travel abroad. The HEV-RNA resulted to be genotype 3 [167].

A swine owner in France was reported to have been contaminated by his pet swine; the pig urinated and defecated outside, and the patient regularly changed the litter. The animal often entered the house and was frequently handled by his owner. Diagnosis revealed that he resulted antibody IgM and HEV-RNA positive for genotype 3. This genotype was similar to those of other European isolates but specific to this case, which suggested autochthonous local transmission [168]. Another study compared HEV sequences identified in 106 patients with sequences isolated from 43 swine. Phylogenetic analysis showed high similarity between human and swine population. The main genotypes were 3c, 3e and 3f [169].

Between 1997 and 2005 in France, seven patients with encephalopathy were diagnosed with acute HEV. The HEV-RNA was sequenced and all isolated belonged to genotype 3 [149].

Another French study investigated 38 cases of HEV using the serological test (the virus was quantified and genotyped), and found that 58% (22/38) patients developed chronic infection. All samples were genotype 3c, 3e, and 3f [170].

A human case of acute HEV infection with genotypes 3c and e were demonstrated in a French kidney transplant recipient. The patient had not recently travelled abroad but reported having eaten both uncooked and cooked figatelli. The two viral sequences were nearly identical to sequences recovered from figatelli [171].

Acute HEV hepatitis related to genotype 4 was observed in a French woman with immune-suppression due to leukemia. This genotype 4 was considered autochthonous since the woman had not previously been to any endemic countries [145].

Fulminant hepatitis occurred in a 73 year old woman with leukemia in France. This patient’s general health conditions deteriorated, and despite ribavirin treatment (600 mg twice daily), she died. The HEV sequence obtained by serum samples belonged to genotype 3f, and the only risk factor for virus transmission was liver sausage (figatelli) consumption [172].

Figatelli consumption was responsible for another outbreak of acute HEV hepatitis in France. The patients were infected with genotypes 3c, 3e, 3f showing great similarity with the strains isolated in pigs [173].

A study cohort was conducted in Austria on 468 adult lung transplant recipients. The samples were tested only for HEV-RNA, and 2.1% of them resulted positive for HEV genotype 3 (median age 40 years) [174].

In Spain, 3/11 cases were confirmed as acute HEV infection. Three different HEV strains were found in the serum of the patients; two were autochthonous genotype 3 and one genotype 1 was imported from Africa [24].

In different regions of Spain, viral genome fragments were amplified from 13 RNA serum samples. The analysis of the sequences led to identifying six genotype 1a (belonging to travelers from India and Bangladesh), six subtype 3f (who did not travel abroad recently) and one genotype 4 viral strain (from a traveler who had returned from Vietnam). Genotype 3 subtype f is responsible for locally acquired HEV infection in different regions of Spain [175].

In Europe, the first case report of HEV in a pregnant woman was identified in Southeast France; the woman was infected with subtype 3f [176].

In addition, a case report in Spain described a case of fulminant HEV hepatitis in a woman taking contraceptive medication who was admitted to a hospital for a liver transplant evaluation. This woman was a 37 year old office worker with no history of alcohol consumption and no close contact with animals. She presented asthenia epigastralgia, nausea and a five-day evolution of fever. The diagnosis was based on the presence of anti-HEV IgM and IgG in serum and confirmed by isolation of a genotype 3f strain [177]. In another study, five cases of fulminant HEV subtype 3f were described in patients with a median age of 49.6 years [178].

**Table 2:** Fulminant hepatitis E cases in Europe.

Country	Number of Patients	Sex	Age	Significant Co-Morbidity	HEV Genotype	Reference
Czech Republic	1	Man	70	Alcohol abuse	3	[165]
France	1	Woman	73	B cell lymphoma	3f	[172]
France	1	Woman	77	Autoimmune hepatitis without hepatocellular failure	3	[167]
France	7	2 Women 5 Men	Range: 50–78	3 Patients with cirrhosis and 2 bridging fibrosis	3	[150]
Spain	1	Woman	37	none	3f	[177]
Spain	5	4 Women 1 Man	Range: 36–78	2 Alcohol abuse and 3 no declared	3f	[178]
Italy	1	Man	79	none	3e	[41]

In 2011, an HEV outbreak in an area of Lazio, Italy, involved five case-patients, who did not travel to disease-endemic areas, so these cases were considered autochthonous. The sequence data from ORF1 and ORF2 regions identified a genotype 4d strain. ORF1 nucleotide sequences from the outbreak showed high similarity among patients and identity with HEV4d swine hb-3 and human T1 isolates in China [59]. Another study, carried out on 43 patients with acute hepatitis symptoms, revealed 39.5% HEV positive cases (17/43): 12 patients harbored genotype 1, which was correlated to travel in endemic areas, and five cases harbored genotype 3 and were autochthonous [50]. Another study characterized the HEV genotypes circulating among the migrant population of Southern Italy [179]. Forty samples from patients not exhibiting symptoms of acute hepatitis (24 men and 16 women), aged between 18 and 47 (mean age: 27.5 years), were tested for HEV-RNA. Only 2/40 (5%) were HEV-RNA positive. The sequenced PCR products belonged to genotypes 1 and 3 [179].

In Milan, Northern Italy, between 1994 and 2009, the phylogenetic analysis based on ORF2 PCR products analysis of 39 viral isolates, belonged to patients with acute HEV who had travelled abroad, showed that they all were infected with a virus of subtype 1a or 1c. On the contrary, ORF2 sequences from five patients with no history of travel abroad, belonged to subtypes 3e, 3f and 3h [107].

Recently, a case of acute fulminant hepatitis E virus was described in Italy. This patient, a 79 year old man, resulted positive to HEV genotype 3e, and it was very similar to a German strain with an identity of 99.2% [41].

Instead, a man, who contracted HEV infection by consumption of figatelli, harbored subtype 3i [180].

Several HEV sequences isolated from humans in different European countries are listed in Table 3.

## 7. Treatment of HEV Infection

Acute hepatitis E usually follows a self-limiting course which only requires symptomatic treatment; however, it is not uncommon for it to take on a severe acute form with the rapid development of acute fulminant hepatitis [181] or it may cause chronic hepatitis. In addition to supportive care, severe acute hepatitis E and fulminant hepatitis as well as chronic E hepatitis may require treatment with an antiviral agent [182,183,184].

Ribavirin has been found to be efficacious in the treatment of chronic hepatitis E in the cases of solid organ transplant recipients, including renal, lung, heart, and pancreas transplant patients. Ribavirin monotherapy has also been found to be beneficial in the treatment of severe acute hepatitis E in both immune-competent and immune-compromised patients with prompt viral clearance and rapidly improved liver function [182,183,184].

Ribavirin therapy is contraindicated in pregnancy owing to teratogenicity, however the risks HEV transmission from an untreated mother to fetus are high, therefore treatment may be offered. Furthermore, available evidence regarding ribavirin administration during pregnancy shows no adverse maternal and fetal outcomes [185,186].

A daily dose of 600 mg of ribavirin has been found to be satisfactory in most cases of hepatitis E treatment. It has been observed that a dose reduction, due to treatment related side effects, such as anemia, is associated with viral rebound and resistance [183,187]. However, when ribavirin was used in conjunction with reduced tacrolimus immunosuppressive therapy and valganciclovir, HEV clearance occurred in a liver-transplanted patient co-infected with herpes virus-6 [188]. Other studies confirmed that after a lower dose of tacrolimus immunosuppressive therapy, liver transplant recipients with chronic hepatitis E benefited most when treated with pegylated interferon for about 3 to 12 months [189,190].

Recently, co-infection with hepatitis E in HIV patients has been observed and the co-infected subjects underwent ribavirin monotherapy treatment [191,192] or pegylated interferon alone [193], furthermore they could be treated with a combination therapy with ribavirin and pegylated interferon [194].

## 8. Conclusions

The data described in this review suggest that hepatitis E, which over previous decades was considered to be an endemic infection prevalently in low-income countries, is a widespread infection, even in industrialized countries. In Europe, the anti-HEV antibody seroprevalence varies greatly, depending on the geographic area considered and the populations studied, as well as the assay used in anti-HEV IgG detection.

For example, anti-HEV IgG prevalence in blood donors ranged from 1.3% (DIAPRO assay, Italy) [108] to 52% (France, Wantai assay) [77]. Contact with pigs or consumption of uncooked pork meat are objective risk factors for transmission of infection, given the high seroprevalence observed in pig veterinarians, pig farmers [88,92,96,101,119], and in the populations that usually consume uncooked pork [59,195]. In fact, in these populations the anti-HEV antibody prevalence was always higher than that observed in control groups, even though they did not reach to 51%. Moreover, the Wantai assay was not used in any of these risk group studies.

Data from literature have demonstrated that the serological assays have a wide range of variability in terms of both sensitivity and specificity [196,197,198,199,200,201]. The results obtained in different comparison analyses were not always concordant. For example, the Wantai assay seems to be able to detect a higher number of previous HEV infections in several studies [197,200], but this evidence has been questioned by other authors [202]. This could be due to different variants or subtypes circulating in the European countries in which the different assays show a different sensitivity. Sensitivity assessment on different HEV subtypes could help in understanding the discrepancies of seroprevalence evaluation by using different kits.

Overall, genotype 3e human subtype is the more frequently described in many European countries (Czech Republic, Germany, France, Greece, Holland, UK), whereas 3f is very common in France, Czech Republic, and Spain. Genotypes 3a and g are less diffused. However the data should be considered as preliminary, since most of the human HEV infections are reported in literature as a genotype without specifying the subtypes.

The several cases of human HEV infections, which are related to food consumption [180,202,203,204], emphasize the relevance of a recent report where the European Food Safety Authority’s Biohazard experts declared that more studies on HEV circulation are necessary to clarify farm-to-table risk assessments [205].

Moreover, the high rate of asymptomatic HEV infections worldwide has led to concern about infection via blood donation. Several authors evidenced that the virus could be transmitted by transfusion in industrialized countries in Europe [206], and genotype 3f was the most related to HEV transmission by blood donation [207].

This review also highlights the increasing number of cases of fulminant hepatitis related to genotype 3 and the status of patient immune-suppression.

**Table 3:** Some representative Human HEV sequences described in European countries.

Country	HEV Genotype	HEV Region (Open Reading Frame)	GenBank Accession Number	Reference
Finland	1	1	FJ890338 to FJ890342	[80]
England and Wales	3	2	AJ879566 to AJ879574 AY362357 AY582797	[155]
	1			
Germany	3	1	AJ889194	[158]
		2	AJ889196	
	3	1	JQ863406 to JQ863418	[163]
	3	1	HE605115	[161]
		2	HE605117	
	3	1	EU879112 GQ266391	[159]
	4	3	JN257704 JN257711	[148]
The Netherlands	3	2	JK645320 to JK645333 KC223601 JX645320 JX645333 JX645334 JX645340 JX678984 KC223601	[94]
			AB385842 to AB385852 DQ200273 to DQ200295	[75]
Czech Republic	3	1	GU299812 GU299813 GU299815 KC924921 to KC924927	[165]
	1		KC924929 to KC924938 KC924928	
	3	1	GU299812 GU299813 GU299815 GU299817	[164]
France	3	2	FJ951641 GQ427003	[173]
			AF110390	[95]
			EU116340	[64]
			EU116336	[166]
			EF028801 EF028802	[207]
	1	1	EF514587	[168]
		2	EF050798	
	4	2	KC437301 to KC437308 GU982294	[147]
	4 3	1	JN794589 JN794587	[146]
	4	2	GU982294	[145]
	3	2	JF730329 to JF730340	[169]
	3	2	GQ427015 to GQ427018 GQ427002 GQ427003 GQ426987 GQ426988 GQ426992 EU116338 EU116340 JQ697492 JQ697493 JF900626 JF900628 JF900630 FJ951640	[136]
	3	2	HQ688787 HQ682232	[34]
	3	1 2	KF921518 KF921517	[167]
	3	2	KM887852 to KM887862	[172]
	3	2	FJ665422 FJ665426 FJ665429	[189]
Hungary	3	2	EF530659 EF530676 EU057982 AY940427	[102]
Spain	3	1	AY570904	[24]
	3	2	AY540113 to AY540115	
	4	1	AY570905	
	3	1	FJ464731	[175]
	3	1	FJ464732	
	4	1	FJ464733	
	1	1	FJ464734	
	3	1	FJ464735	
	1	1	FJ464736	
	3	1	FJ464737	
	3	1	FJ464738	
	1	1	FJ464739 to FJ464743	
	3	2	FJ464744 to FJ464746	
	1	2	FJ464747	
	3	2	FJ464748	
	3	2	FJ464749	
	1	2	FJ464750	
	1	2	FJ464751	
	3	2	FJ464752	
Italy	1	2	HM446588 to HM446631	[107]
	4	2	JX401928	[59]
	3	1	JX898218 JX898219	[179]
	3	1	FJ998019	[41]

## References

[1] Purcell R.H., Emerson S.U. (2008). Hepatitis E: An emerging awareness of an old disease. J. Hepatol..

[2] Rein D.B., Stevens G.A., Theaker J., Wittenborn J.S., Wiersma S.T. (2012). The global burden of hepatitis E virus genotypes 1 and 2 in 2005. Hepatology.

[3] Guthmann J.P., Klovstad H., Boccia D., Hamid N., Pinoges L., Nizou J.Y., Tatay M., Diaz F., Moren A., Grais R.F. (2006). A large outbreak of hepatitis E among a displaced population in Darfur, Sudan, 2004: The role of water treatment methods. Clin. Infect. Dis..

[4] Kumar S., Subhadra S., Singh B., Panda B.K. (2013). Hepatitis E virus: The current scenario. Int. J. Infect. Dis..

[5] Nicand E., Bigaillon C., Tessé S. (2009). Hepatitis E: An emerging disease?. Pathol. Biol..

[6] Balayan M.S., Andjaparidze A.G., Savinskaya S.S., Ketiladze E.S., Braginsky D.M., Savinov A.P., Poleschuk V.F. (1983). Evidence for a virus in non-A, non-B hepatitis transmitted via the fecal-oral route. Intervirology.

[7] Arankalle V.A., Chadha M.S., Tsarev S.A., Emerson S.U., Risbud A.R., Banerjee K., Purcell R.H. (1994). Seroepidemiology of water-borne hepatitis in India and evidence for a third enterically transmitted hepatitis agent. Proc. Natl. Acad. Sci. USA.

[8] Emerson S.U., Anderson D., Arankalle V.A., Meng X.J., Purdy M., Schlauder G.G., Tsarev S.A. (2004). Virus Taxonomy: VII-Ith Report of the ICTV.

[9] Sun Z.F., Larsen C.T., Huang F.F., Billam P., Pierson F.W., Toth T.E., Meng X.J. (2004). Generation and infectivity titration of an infectious stock of avian hepatitis E virus (HEV) in chickens and cross-species infection of turkeys with avian HEV. J. Clin. Microbiol..

[10] Tam A.W., Smith M.M., Guerra M.E., Huang C.C., Bradley D.W., Fry K.E., Reyes G.R. (1991). Hepatitis E virus (HEV): Molecular cloning and sequencing of the full-length viral genome. Virology.

[11] Panda S.K., Thakral D., Rehman S. (2007). Hepatitis E virus. Rev. Med. Virol..

[12] Mushahwar I.K. (2008). Hepatitis E virus: Molecular virology, clinical features, diagnosis, transmission, epidemiology, and prevention. J. Med. Virol..

[13] Ahmad I., Holla R.P., Jameel S. (2011). Molecular virology of hepatitis E virus. Virus Res..

[14] Tsega E., Krawczynski K., Hansson B.G., Nordenfelt E. (1993). Hepatitis E virus infection in pregnancy in Ethiopia. Ethiop. Med. J..

[15] Van Cuyck-Gandré H., Zhang H.Y., Tsarev S.A., Warren R.L., Caudill J.D., Snellings N.J., Bégot L., Innis B.L., Longer C.F. (2000). Short report: Phylogenetically distinct hepatitis E viruses in Pakistan. Am. J. Trop. Med. Hyg..

[16] Drabick J.J., Gambel J.M., Gouvea V.S., Caudill J.D., Sun W., Hoke C.H., Innis B.L. (1997). A cluster of acute hepatitis E infection in United Nations Bangladeshi peacekeepers in Haiti. Am. J. Trop. Med. Hyg..

[17] Alavian S.M. (2010). A look at the past history of hepatitis E in Haiti: Should it be a warning sign during the current crisis?. Hepat. Mon..

[18] Villalba M.C.M., Lay L.A.R., Chandra V., Corredor M.B., Frometa S.S., Moreno A.G., Jameel S. (2008). Hepatitis E virus genotype 1, Cuba. Emerg. Infect. Dis..

[19] Chatterjee R., Tsarev S., Pillot J., Coursaget P., Emerson S.U., Purcell R.H. (1997). African strains of hepatitis E virus that are distinct from Asian strains. J. Med. Virol..

[20] Chandra N.S., Sharma A., Rai R.R., Malhotra B. (2012). Contribution of hepatitis E virus in acute sporadic hepatitis in north western India. Indian J. Med. Res..

[21] Usmanov R.K., Favorov M.O., Vasil’eva V.I., Aĭdarbekova D.S., Karas’ F.R., Iashina T.L., Mineeva R.M., Aslanian R.G., Zairov G.K., Alymbaeva D.B. (1991). A comparative study of enteral hepatitis E (non-A, non-B) in the valley and mountainous areas of Kirghizia. Vopr. Virusol..

[22] Meng J., Cong M., Dai X., Pillot J., Purdy M.A., Fields H.A., Khudyakov Y.E. (1999). Primary structure of open reading frame 2 and 3 of the hepatitis E virus isolated from Morocco. J. Med. Virol..

[23] Van Cuyck-Gandré H., Zhang H.Y., Tsarev S.A., Clements N.J., Cohen S.J., Caudill J.D., Buisson Y., Coursaget P., Warren R.L., Longer C.F. (1997). Characterization of hepatitis Evirus (HEV) from Algeria and Chad by partial genome sequence. J. Med. Virol..

[24] Buti M., Clemente-Casares P., Jardi R., Formiga-Cruz M., Schaper M., Valdes A., Rodriguez-Frias F., Esteban R., Girones R. (2004). Sporadic cases of acute autochthonous hepatitis E in Spain. J. Hepatol..

[25] Huang C.C., Nguyen D., Fernandez J., Yun K.Y., Fry K.E., Bradley D.W., Tam A.W., Reyes G.R. (1992). Molecular cloning and sequencing of the Mexico isolate of hepatitis E virus (HEV). Virology.

[26] Buisson Y., Grandadam M., Nicand E., Cheval P., van Cuyck-Gandre H., Innis B., Rehel P., Coursaget P., Teyssou R., Tsarev S. (2000). Identification of a novel hepatitis E virus in Nigeria. J. Gen. Virol..

[27] Lu L., Li C., Hagedorn C.H. (2006). Phylogenetic analysis of global hepatitis E virus sequences: Genetic diversity, subtypes and zoonosis. Rev. Med. Virol..

[28] Kwo P.Y., Schlauder G.G., Carpenter H.A., Murphy P.J., Rosenblatt J.E., Dawson G.J., Mast E.E., Krawczynski K., Balan V. (1997). Acute hepatitis E by a new isolate acquired in the United States. Mayo Clin. Proc..

[29] Schlauder G.G., Dawson G.J., Erker J.C., Kwo P.Y., Knigge M.F., Smalley D.L., Rosenblatt J.E., Desai S.M., Mushahwar I.K. (1998). The sequence and phylogenetic analysis of a novel hepatitis E virus isolated from a patient with acute hepatitis reported in the United States. J. Gen. Virol..

[30] Huang F.F., Haqshenas G., Guenette D.K., Halbur P.G., Schommer S.K., Pierson F.W., Toth T.E., Meng X.J. (2002). Detection by reverse transcription-PCR and genetic characterization of field isolates of swine hepatitis E virus from pigs in different geographic regions of the United States. J. Clin. Microbiol..

[31] Takahashi M., Nishizawa T., Yoshikawa A., Sato S., Isoda N., Ido K., Sugano K., Okamoto H. (2002). Identification of two distinct genotypes of hepatitis E virus in a Japanese patient with acute hepatitis who had not travelled abroad. J. Gen. Virol..

[32] Ahn J.M., Kang S.G., Lee D.Y., Shin S.J., Yoo H.S. (2005). Identification of novel human hepatitis E virus (HEV) isolates and determination of the seroprevalence of HEV in Korea. J. Clin. Microbiol..

[33] Van der Poel W.H., Verschoor F., van der Heide R., Herrera M.I., Vivo A., Kooreman M., de Roda Husman A.M. (2001). Hepatitis E virus sequences in swine related to sequences in humans, The Netherlands. Emerg. Infect. Dis..

[34] Renou C., Pariente A., Cadranel J.F., Nicand E., Pavio N. (2011). Clinically silent forms may partly explain the rarity of acute cases of autochthonous genotype 3c hepatitis E infection in France. J. Clin. Virol..

[35] Rose N., Lunazzi A., Dorenlor V., Merbah T., Eono F., Eloit M., Madec F., Pavio N. (2011). High prevalence of Hepatitis E virus in French domestic pigs. Comp. Immunol. Microbiol. Infect. Dis..

[36] Wenzel J.J., Preiss J., Schemmerer M., Huber B., Plentz A., Jilg W. (2011). Detection of hepatitis E virus (HEV) from porcine livers in Southeastern Germany and high sequence homology to human HEV isolates. J. Clin. Virol..

[37] Di Bartolo I., Ponterio E., Castellini L., Ostanello F., Ruggeri F.M. (2011). Viral and antibody HEV prevalence in swine at slaughter house in Italy. Vet. Microbiol..

[38] Wu J.C., Chen C.M., Chiang T.Y., Tsai W.H., Jeng W.J., Sheen I.J., Lin C.C., Meng X.J. (2002). Spread of hepatitis E virus among different-aged pigs: Two-year survey in Taiwan. J. Med. Virol..

[39] Banks M., Bendall R., Grierson S., Heath G., Mitchell J., Dalton H. (2004). Human and porcine hepatitis E virus strains, United Kingdom. Emerg. Infect. Dis..

[40] Zanetti A.R., Schlauder G.G., Romanò L., Tanzi E., Fabris P., Dawson G.J., Mushahwar I.K. (1999). Identification of a novel variant of hepatitis E virus in Italy. J. Med. Virol..

[41] Festa S., Garbuglia A.R., Baccini F., Panzuto F., Capobianchi M.R., Santino I., Purchiaroni F., Orgera G., Delle Fave G., Marignani M. (2014). Acute fulminant hepatitis E virus genotype 3e infection: description of the first case in Europe. Scand. J. Infect. Dis..

[42] Schlauder G.G., Desai S.M., Zanetti A.R., Tassopoulos N.C., Mushahwar I.K. (1999). Novel hepatitis E virus (HEV) isolates from Europe: Evidence for additional genotypes of HEV. J. Med. Virol..

[43] Clemente-Casares P., Pina S., Buti M., Jardi R., Martin M., Bofill-Mas S., Girones R. (2003). Hepatitis E virus epidemiology in industrialized countries. Emerg. Infect. Dis..

[44] Sato Y., Sato H., Naka K., Furuya S., Tsukiji H., Kitagawa K., Sonoda Y., Usui T., Sakamoto H., Yoshino S. (2011). A nationwide survey of hepatitis E virus (HEV) infection in wild boars in Japan: Identification of boar HEV strains of genotypes 3 and 4 and unrecognized genotypes. Arch. Virol..

[45] Wiratsudakul A., Sariya L., Prompiram P., Tantawet S., Suraruangchai D., Sedwisai P., Sangkachai N., Suksai P., Ratanakorn P. (2012). Detection and phylogenetic characterization of hepatitis E virus genotype 3 in a captive wild boar in Thailand. J. Zoo Wildl. Med..

[46] Keawcharoen J., Thongmee T., Panyathong R., Joiphaeng P., Tuanthap S., Oraveerakul K., Theamboonlers A., Poovorawan Y. (2013). Hepatitis E virus genotype 3f sequences from pigs in Thailand, 2011–2012. Virus Genes.

[47] Kaba M., Davoust B., Cabre O., Colson P. (2011). Hepatitis E virus genotype 3f in pigs in New Caledonia. Aust. Vet. J..

[48] Lu L., Drobeniuc J., Kobylnikov N., Usmanov R.K., Robertson B.H., Favorov M.O., Margolis H.S. (2004). Complete sequence of a Kyrgyzstan swine hepatitis E virus (HEV) isolated from a piglet thought to be experimentally infected with human HEV. J. Med. Virol..

[49] Martelli F., Toma S., di Bartolo I., Caprioli A., Ruggeri F.M., Lelli D., Bonci M., Ostanello F. (2010). Detection of hepatitis E virus (HEV) in Italian pigs displaying different pathological lesions. Res. Vet. Sci..

[50] La Rosa G., Muscillo M., Vennarucci V.S., Garbuglia A.R., la Scala P., Capobianchi M.R. (2011). Hepatitis E virus in Italy: Molecular analysis of travel-related and autochthonous cases. J. Gen. Virol..

[51] Mirazo S., Ramos N., Russi J.C., Arbiza J. (2013). Genetic heterogeneity and subtyping of human hepatitis E virus isolates from Uruguay. Virus Res..

[52] Garkavenko O., Obriadina A., Meng J., Anderson D.A., Benard H.J., Schroeder B.A., Khudyakov Y.E., Fields H.A., Croxson M.C. (2001). Detection and characterisation of swine hepatitis E virus in New Zealand. J. Med. Virol..

[53] Schlauder G.G., Frider B., Sookoian S., Castaño G.C., Mushahwar I.K. (2000). Identification of 2 novel isolates of hepatitis E virus in Argentina. J. Infect. Dis..

[54] Dell’Amico M.C., Cavallo A., Gonzales J.L., Bonelli S.I., Valda Y., Pieri A., Segund H., Ibañez R., Mantella A., Bartalesi F. (2011). Hepatitis E virus genotype 3 in humans and Swine, Bolivia. Emerg. Infect. Dis..

[55] Worm H.C., Schlauder G.G., Wurzer H., Mushahwar I.K. (2000). Identification of a novel variant of hepatitis E virus in Austria: Sequence, phylogenetic and serological analysis. J. Gen. Virol..

[56] Moal V., Gérolami R., Ferretti A., Purgus R., Devichi P., Burtey S., Colson P. (2014). Hepatitis E virus of subtype 3i in chronically infected kidney transplant recipients in southeastern France. J. Clin. Microbiol..

[57] Pei Y., Yoo D. (2002). Genetic characterization and sequence heterogeneity of a canadian isolate of Swine hepatitis E virus. J. Clin. Microbiol..

[58] Ward P., Müller P., Letellier A., Quessy S., Simard C., Trottier Y.L., Houde A., Brassard J. (2008). Molecular characterization of hepatitis E virus detected in swine farms in the province of Quebec. Can. J. Vet. Res..

[59] Garbuglia A.R., Scognamiglio P., Petrosillo N., Mastroianni C.M., Sordillo P., Gentile D., La Scala P., Girardi E., Capobianchi M.R. (2013). Hepatitis E virus genotype 4 outbreak, Italy, 2011. Emerg. Infect. Dis..

[60] Monne I., Ceglie L., di Martino G., Natale A., Zamprogna S., Morreale A., Rampazzo E., Cattoli G., Bonfanti L. (2015). Hepatitis E virus genotype 4 in a pig farm, Italy, 2013. Epidemiol. Infect..

[61] Wichmann O., Schimanski S., Koch J., Kohler M., Rothe C., Plentz A., Jilg W., Stark K. (2008). Phylogenetic and case-control study on hepatitis E virus infection in Germany. J. Infect. Dis..

[62] Liu Z., Chi B., Takahashi K., Mishiro S. (2003). A genotype IV hepatitis E virus strain that may be indigenous to Changchun, China. Intervirology.

[63] Chau T.N., Lai S.T., Tse C., Ng T.K., Leung V.K., Lim W., Ng M.H. (2006). Epidemiology and clinical features of sporadic hepatitis E as compared with hepatitis A. Am. J. Gastroenterol..

[64] Gérolami R., Moal V., Colson P. (2008). Chronic hepatitis E with cirrhosis in a kidney-transplant recipient. N. Engl. J. Med..

[65] Dalton H.R., Fellows H.J., Stableforth W., Joseph M., Thurairajah P.H., Warshow U., Hazeldine S., Remnarace R., Ijaz S., Hussaini S.H. (2007). The role of hepatitis E virus testing in drug-induced liver injury. Aliment. Pharmacol. Ther..

[66] Said B., Ijaz S., Kafatos G., Booth L., Thomas H.L., Walsh A., Ramsay M., Morgan D. (2009). Hepatitis E Incident Investigation Team. Hepatitis E outbreak on cruise ship. Emerg. Infect. Dis..

[67] Kamar N., Bendall R.P., Peron J.M., Cintas P., Prudhomme L., Mansuy J.M., Rostaing L., Keane F., Ijaz S., Izopet J. (2011). Hepatitis E virus and neurologic disorders. Emerg. Infect. Dis..

[68] Kamar N., Izopet J., Cintas P., Garrouste C., Uro-Coste E., Cointault O., Rostaing L. (2010). Hepatitis E virus-induced neurological symptoms in a kidney-transplant patient with chronic hepatitis. Am. J. Transplant..

[69] Tse A.C., Cheung R.T., Ho S.L., Chan K.H. (2012). Guillain-Barré syndrome associated with acute hepatitis E infection. J. Clin. Neurosci..

[70] Kamar N., Garrouste C., Haagsma E.B., Garrigue V., Pischke S., Chauvet C., Dumortier J., Cannesson A., Cassuto-Viguier E., Thervet E. (2011). Factors associated with chronic hepatitis in patients with hepatitis E virus infection who have received solid organ transplants. Gastroenterology.

[71] Herremans M., Vennema H., Bakker J., van der Veer B., Duizer E., Benne C.A., Waar K., Hendrixks B., Schneeberger P., Blaauw G. (2007). Swine-like hepatitis E viruses are a cause of unexplained hepatitis in the Netherlands. J. Viral. Hepat..

[72] Waar K., Herremans M.M., Vennema H., Koopmans M.P., Benne C.A. (2005). Hepatitis E is a cause of unexplained hepatitis in The Netherlands. J. Clin. Virol..

[73] Baylis S.A., Gärtner T., Nick S., Ovemyr J., Blümel J. (2012). Occurrence of hepatitis E virus RNA in plasma donations from Sweden, Germany and the United States. Vox Sang..

[74] Ijaz S., Szypulska R., Tettmar K.I., Kitchen A., Tedder R.S. (2012). Detection of hepatitis E virus RNA in plasma mini-pools from blood donors in England. Vox Sang..

[75] Fogeda M., Avellón A., Echevarría J.M. (2012). Prevalence of specific antibody to hepatitis E virus in the general population of the community of Madrid, Spain. J. Med. Virol..

[76] Bura M., Michalak M., Chojnicki M., Czajka A., Kowala-Piaskowska A., Mozer-Lisewska I. (2015). Seroprevalence of anti-HEV IgG in 182 Polish patients. Postep. Hig. Med. Dosw..

[77] Mansuy J.M., Bendall R., Legrand-Abravanel F., Sauné K., Miédouge M., Ellis V., Rech H., Destruel F., Kamar N., Dalton H.R. (2011). Hepatitis E virus antibodies in blood donors, France. Emerg. Infect. Dis..

[78] Norder H., Sundqvist L., Magnusson L., Østergaard Breum S., Löfdahl M., Larsen L.E., Hjulsager C.K., Magnius L., Böttiger B.E., Widén F. (2009). Endemic hepatitis E in two Nordic countries. Euro. Surveill..

[79] Olsen B., Axelsson-Olsson D., Thelin A., Weiland O. (2006). Unexpected high prevalence of IgG-antibodies to hepatitis E virus in Swedish pig farmers and controls. Scand. J. Infect. Dis..

[80] Kantala T., Maunula L., von Bonsdorff C.H., Peltomaa J., Lappalainen M. (2009). Hepatitis E virus in patients with unexplained hepatitis in Finland. J. Clin. Virol..

[81] Dalton H.R., Stableforth W., Thurairajah P., Hazeldine S., Remnarace R., Usama W., Farrington L., Hamad N., Sieberhagen C., Ellis V. (2008). Autochthonous hepatitis E in Southwest England: Natural history, complications and seasonal variation, and hepatitis E virus IgG seroprevalence in blood donors, the elderly and patients with chronic liver disease. Eur. J. Gastroenterol. Hepatol..

[82] Cleland A., Smith L., Crossan C., Blatchford O., Dalton H.R., Scobie L., Petrik J. (2013). Hepatitis E virus in Scottish blood donors. Vox Sang..

[83] Christensen P.B., Engle R.E., Hjort C., Homburg K.M., Vach W., Georgsen J., Purcell R.H. (2008). Time trend of the prevalence of hepatitis E antibodies among farmers and blood donors: A potential zoonosis in Denmark. Clin. Infect. Dis..

[84] Christensen P.B., Engle R.E., Jacobsen S.E., Krarup H.B., Georgsen J., Purcell R.H. (2002). High prevalence of hepatitis E antibodies among Danish prisoners and drug users. J. Med. Virol..

[85] Van Hoecke F., van Maerken T., de Boulle M., Geerts A., Vlierberghe V., Colle I., Padalko H.E. (2012). Hepatitis E seroprevalence in east and west Flanders, Belgium. Acta Gastroenterol. Belg..

[86] Abe K., Hayakawa E., Sminov A.V., Rossina A.L., Ding X., Huy T.T., Sata T., Uchaikin V.F. (2004). Molecular epidemiology of hepatitis B, C, D and E viruses among children in Moscow, Russia. J. Clin. Virol..

[87] Faber M.S., Wenzel J.J., Jilg W., Thamm M., Höhle M., Stark K. (2012). Hepatitis E virus seroprevalence among adults, Germany. Emerg. Infect. Dis..

[88] Juhl D., Baylis S.A., Blümel J., Görg S., Hennig H. (2014). Seroprevalence and incidence of hepatitis E virus infection in German blood donors. Transfusion.

[89] Dremsek P., Wenzel J.J., Johne R., Ziller M., Hofmann J., Groschup M.H., Werdermann S., Mohn U., Dorn S., Motz M. (2012). Seroprevalence study in forestry workers from eastern Germany using novel genotype 3- and rat hepatitis E virus-specific immunoglobulin G ELISAs. Med. Microbiol. Immunol..

[90] Drobeniuc J., Favorov M.O., Shapiro C.N., Bell B.P., Mast E.E., Dadu A., Culver D., Iarovoi P., Robertson B.H., Margolis H.S. (2001). Hepatitis E virus antibody prevalence among persons who work with swine. J. Infect. Dis..

[91] Dlhý J., Benes C. (2007). Imported viral hepatitis in the Czech Republic. Klin. Mikrobiol. Infekc. Lek..

[92] Pazdiora P., Nĕmecek V., Topolcan O. (1996). Initial results of monitoring hepatitis E virus antibodies in selected population groups in the West Bohemia Region. Preliminary report. Epidemiol. Mikrobiol. Imunol..

[93] Bouwknegt M., Engel B., Herremans M.M., Widdowson M.A., Worm H.C., Koopmans M.P., Frankena K., de Roda Husman A.M., de Jong M.C., van der Poel W.H. (2008). Bayesian estimation of hepatitis E virus seroprevalence for populations with different exposure levels to swine in The Netherlands. Epidemiol. Infect..

[94] Slot E., Hogema B.M., Riezebos-Brilman A., Kok T.M., Molier M., Zaaijer H.L. (2013). Silent hepatitis E virus infection in Dutch blood donors, 2011 to 2012. Euro Surveill..

[95] Mansuy J.M., Legrand-Abravanel F., Calot J.P., Peron J.M., Alric L., Agudo S., Rech H., Destruel F., Izopet J. (2008). High prevalence of anti-hepatitis E virus antibodies in blood donors from South West France. J. Med. Virol..

[96] Mansuy J.M., Peron J.M., Abravanel F., Poirson H., Dubois M., Miedouge M., Vischi F., Alric L., Vinel J.P., Izopet J. (2004). Hepatitis E in the south west of France in individuals who have never visited an endemic area. J. Med. Virol..

[97] Carpentier A., Chaussade H., Rigaud E., Rodriguez J., Berthault C., Boué F., Tognon M., Touzé A., Garcia-Bonnet N., Choutet P. (2012). High hepatitis E virus seroprevalence in forestry workers and in wild boars in France. J. Clin. Microbiol..

[98] Boutrouille A., Bakkali-Kassimi L., Crucière C., Pavio N. (2007). Prevalence of anti-hepatitis E virus antibodies in French blood donors. J. Clin. Microbiol..

[99] Lagler H., Poeppl W., Winkler H., Herkner H., Faas A., Mooseder G., Burgmann H. (2014). Hepatitis E virus seroprevalence in Austrian adults: A nationwide cross-sectional study among civilians and military professionals. PLoS ONE.

[100] Kaufmann A., Kenfak-Foguena A., André C., Canellini G., Bürgisser P., Moradpour D., Darling K.E., Cavassini M. (2011). Hepatitis E virus seroprevalence among blood donors in southwest Switzerland. PLoS ONE.

[101] Voiculescu M., Iliescu L., Ionescu C., Micu L., Ismail G., Zilisteanu D., Radasan A., Micu G., Pertache I. (2010). A cross-sectional epidemiological study of HBV, HCV, HDV and HEV prevalence in the Sub Carpathian and South-Eastern regions of Romania. J. Gastrointestin. Liver Dis..

[102] Reuter G., Fodor D., Forgách P., Kátai A., Szucs G. (2009). Characterization and zoonotic potential of endemic hepatitis E virus (HEV) strains in humans and animals in Hungary. J. Clin. Virol..

[103] Tarrago D., López-Vélez R., Turrientes C., Baquero F., Mateos M.L. (2000). Prevalence of hepatitis E antibodies in immigrants from developing countries. Eur. J. Clin. Microbiol. Infect. Dis..

[104] Echevarría J.M., Fogeda M., Avellón A. (2011). Diagnosis of acute hepatitis E by antibody and molecular testing: A study on 277 suspected cases. J. Clin. Virol..

[105] Macedo G., Pinto T., Sarmento J.A., Vale A.M., Ribeiro T. (1998). The first assessment of hepatitis E virus seroprevalence in northern Portugal. Acta Med. Port..

[106] Duque V., Ventura C., Seixas D., Da Cunha S., Meliço-Silvestre A. (2012). First report of acute autochthonous hepatitis E in Portugal. J. Infect. Dev. Ctries..

[107] Romanò L., Paladini S., Tagliacarne C., Canuti M., Bianchi S., Zanetti A.R. (2011). Hepatitis E in Italy: A long-term prospective study. J. Hepatol..

[108] Scotto G., Martinelli D., Centra M., Querques M., Vittorio F., Delli Carri P., Tartaglia A., Campanale F., Bulla F., Prato R. (2014). Epidemiological and clinical features of HEV infection: A survey in the district of Foggia (Apulia, SouthernItaly). Epidemiol. Infect..

[109] De Donno A., Chironna M., Craca R., Paiano A., Zizza A., Guido M., Carrozzini F., Germinario C., Gabutti G. (2003). Anti-HEV seroprevalence in the area of Lecce. Ann. Ig..

[110] Cacciola I., Messineo F., Cacopardo B., di Marco V., Galli C., Squadrito G., Musolino C., Saitta C., Pollicino T., Raimondo G. (2011). Hepatitis E virus infection as a cause of acute hepatitis in Southern Italy. Dig. Liver Dis..

[111] Lanini S., Garbuglia A.R., Lapa D., Puro V., Navarra A., Pergola C., Ippolito G., Capobianchi M.R. (2015). Epidemiology of HEV in the Mediterranean basin: 10-year prevalence in Italy. BMJ Open.

[112] Petrović T., Lupulović D., Jiménez de Oya N., Vojvodić S., Blázquez A.B., Escribano-Romero E., Martín-Acebes M.A., Potkonjak A., Milošević V., Lazić S. (2014). Prevalence of hepatitis E virus (HEV) antibodies in Serbian blood donors. J. Infect. Dev. Ctries..

[113] Kondili L.A., Chionne P., Porcaro A., Madonna E., Taffon S., Resuli B., Taliani G., Rapicetta M. (2006). Seroprevalence of hepatitis E virus (HEV) antibody and the possible association with chronic liver disease: A case-control study in Albania. Epidemiol. Infect..

[114] Dalekos G.N., Zervou E., Elisaf M., Germanos N., Galanakis E., Bourantas K., Siamopoulos K.C., Tsianos E.V. (1998). Antibodies to hepatitis E virus among several populations in Greece: Increased prevalence in an hemodialysis unit. Transfusion.

[115] Cevrioglu A.S., Altindis M., Tanir H.M., Aksoy F. (2004). Investigation of the incidence of hepatitis E virus among pregnant women in Turkey. J. Obstet. Gynaecol. Res..

[116] Oncu S., Oncu S., Okyay P., Ertug S., Sakarya S. (2006). Prevalence and risk factors for HEV infection in pregnant women. Med. Sci. Monit..

[117] Maral I., Budakoglu I.I., Ceyhan M.N., Atak A., Bumin M.A. (2010). Hepatitis E virus seroepidemiology and its change during 1 year in primary school students in Ankara, Turkey. Clin. Microbiol. Infect..

[118] Atabek M.E., Fýndýk D., Gulyuz A., Erkul I. (2004). Prevalence of anti-HAV and anti-HEV antibodies in Konya, Turkey. Health Policy.

[119] Ceylan A., Ertem M., Ilcin E., Ozekinci T. (2003). A special risk group for hepatitis E infection: Turkish agricultural workers who use untreated waste water for irrigation. Epidemiol. Infect..

[120] Dalton H.R., Bendall R.P., Keane F.E., Tedder R.S., Ijaz S. (2009). Persistent carriage of hepatitis E virus in patients with HIV infection. N. Engl. J. Med..

[121] Crum-Cianflone N.F., Curry J., Drobeniuc J., Weintrob A., Landrum M., Ganesan A., Bradley W., Agan B.K., Kamili S. (2012). Hepatitis E virus infection in HIV-infected persons. Emerg. Infect. Dis..

[122] Riveiro-Barciela M., Buti M., Homs M., Campos-Varela I., Cantarell C., Crespo M., Castells L., Tabernero D., Quer J., Esteban R., Rodriguez-Frías F. (2014). Cirrhosis, liver transplantation and HIV infection are risk factors associated with hepatitis E virus infection. PLoS ONE.

[123] Péron J.M., Mansuy J.M., Récher C., Bureau C., Poirson H., Alric L., Izopet J., Vinel J.P. (2006). Prolonged hepatitis E in an immunocompromised patient. J. Gastroenterol. Hepatol..

[124] Kamar N., Mansuy J.M., Cointault O., Selves J., Abravanel F., Danjoux M., Otal P., Esposito L., Durand D., Izopet J. (2008). Hepatitis E virus-related cirrhosis in kidney- and kidney-pancreas-transplant recipients. Am. J. Transplant..

[125] Fabrizi F., Lunghi G., Bacchini G., Corti M., Pagano A., Locatelli F. (1997). Hepatitis E virus infection in haemodialysis patients: A seroepidemiological survey. Nephrol. Dial. Transplant..

[126] Sylvan S.P., Jacobson S.H., Christenson B. (1998). Prevalence of antibodies to hepatitis E virus among hemodialysis patients in Sweden. J. Med. Virol..

[127] Stefanidis I., Zervou E.K., Rizos C., Syrganis C., Patsidis E., Kyriakopoulos G., Sdrakas L., Tsianas N., Rigopoulou E.I., Liakopoulosm V. (2004). Hepatitis E virus antibodies in hemodialysis patients: An epidemiological survey in central Greece. Int. J. Artif. Organs.

[128] Uçar E., Cetin M., Kuvandik C., Helvaci M.R., Güllü M., Hüzmeli C. (2009). Hepatitis E virus seropositivity in hemodialysis patients in Hatay province, Turkey. Mikrobiyol. Bul..

[129] Harrison A., Scobie L., Crossan C., Parry R., Johnston P., Stratton J., Dickinson S., Ellis V., Hunter J.G., Prescott O.R. (2013). Hepatitis E seroprevalence in recipients of renal transplants or haemodialysis in southwest England: A case-control study. J. Med. Virol..

[130] Keane F., Gompels M., Bendall R., Drayton R., Jennings L., Black J., Baragwanath G., Lin N., Henley W., Ngui S.L. (2012). Hepatitis E virus coinfection in patients with HIV infection. HIV Med..

[131] Hassing R.J., van der Eijk A.A., Lopes V.B., Snijdewind I.J., de Man R.A., Pas S.D., van der Ende M.E. (2014). Hepatitis E prevalence among HIV infected patients with elevated liver enzymes in the Netherlands. J. Clin. Virol..

[132] Hoerning A., Hegen B., Wingen A.M., Cetiner M., Lainka E., Kathemann S., Fiedler M., Timm J., Wenzel J.J., Hoyer P.F. (2012). Prevalence of hepatitis E virus infection in pediatric solid organ transplant recipients—A single-center experience. Pediatr. Transplant..

[133] Kenfak-Foguena A., Schöni-Affolter F., Bürgisser P., Witteck A., Darling K.E., Kovari H., Kaiser L., Evison J.M., Elzi L., Gurter-De la Fuente V. (2011). Data Center of the Swiss HIV Cohort Study, Lausanne, Switzerland. Hepatitis E Virus seroprevalence and chronic infections in patients with HIV, Switzerland. Emerg. Infect. Dis..

[134] Kamar N., Selves J., Mansuy J.M., Ouezzani L., Péron J.M., Guitard J., Cointault O., Esposito L., Abravanel F., Danjoux M. (2008). Hepatitis E virus and chronic hepatitis in organ-transplant recipients. N. Engl. J. Med..

[135] Moal V., Motte A., Kaba M., Gerolami R., Berland Y., Colson P. (2013). Hepatitis E virus serological testing in kidney transplant recipients with elevated liver enzymes in 2007–2011 in southeastern France. Diagn. Microbiol. Infect. Dis..

[136] Moal V., Legris T., Burtey S., Morange S., Purgus R., Dussol B., Garcia S., Motte A., Gérolami R., Berland Y. (2013). Infection with hepatitis E virus in kidney transplant recipients in southeastern France. J. Med. Virol..

[137] Maylin S., Stephan R., Molina J.M., Peraldi M.N., Scieux C., Nicand E., Simon F., Delaugerre C. (2012). Prevalence of antibodies and RNA genome of hepatitis E virus in a cohort of French immunocompromised. J. Clin. Virol..

[138] Kaba M., Richet H., Ravaux I., Moreau J., Poizot-Martin I., Motte A., Nicolino-Brunet C., Dignat-George F., Ménard A., Dhiver C. (2011). Hepatitis E virus infection in patients infected with the human immunodeficiency virus. J. Med. Virol..

[139] Renou C., Lafeuillade A., Cadranel J.F., Pavio N., Pariente A., Allègre T., Poggi C., Pénaranda G., Cordier F., Nicand E. (2010). Hepatitis E virus in HIV-infected patients. AIDS.

[140] Scotto G., Grisorio B., Filippini P., Ferrara S., Massa S., Bulla F., Martini S., Filippini A., Tartaglia A., lo Muzio L. (2015). Hepatitis E virus co-infection in HIV-infected patients in Foggia and Naples in southern Italy. Infect. Dis..

[141] Jardi R., Crespo M., Homs M., van den Eynde E., Girones R., Rodriguez-Manzano J., Caballero A., Buti M., Esteban R., Rodriguez-Frias F. (2012). HIV, HEV and cirrhosis: Evidence of a possible link from eastern Spain. HIV Med..

[142] Mateos-Lindemann M.L., Diez-Aguilar M., Galdamez A.L., Galán J.C., Moreno A., Pérez-Gracia M.T. (2014). Patients infected with HIV are at high-risk for hepatitis E virus infection in Spain. J. Med. Virol..

[143] Rivero-Juarez A., Martinez-Dueñas L., Martinez-Peinado A., Camacho A., Cifuentes C., Gordon A., Frias M., Torre-Cisneros J., Pineda J.A., Rivero A. (2015). High hepatitis E virus seroprevalence with absence of chronic infection in HIV-infected patients. J. Infect..

[144] Politou M., Boti S., Androutsakos T., Valsami S., Pittaras T., Kapsimali V. (2015). Seroprevalence of hepatitis E in HIV infected patients in Greece. J. Med. Virol..

[145] Tessé S., Lioure B., Fornecker L., Wendling M.J., Stoll-Keller F., Bigaillon C., Nicand E. (2012). Circulation of genotype 4 hepatitis E virus in Europe: First autochthonous hepatitis E infection in France. J. Clin. Virol..

[146] Colson P., Romanet P., Moal V., Borentain P., Purgus R., Benezech A., Motte A., Gérolami R. (2012). Autochthonous infections with hepatitis E virus genotype 4, France. Emerg. Infect. Dis..

[147] Jeblaoui A., Haim-Boukobza S., Marchadier E., Mokhtari C., Roque-Afonso A.M. (2013). Genotype 4 hepatitis E virus in France: An autochthonous infection with a more severe presentation. Clin. Infect. Dis..

[148] Baylis S.A., Koc O., Nick S., Blümel J. (2012). Widespread distribution of hepatitis E virus in plasma fractionation pools. Vox Sang..

[149] Péron J.M., Bureau C., Poirson H., Mansuy J.M., Alric L., Selves J., Dupuis E., Izopet J., Vinel J.P. (2007). Fulminant liver failure from acute autochthonous hepatitis E in France: Description of seven patients with acute hepatitis E and encephalopathy. J. Viral. Hepat..

[150] Mennecier D., Nicand E., Grandadam M., Bronstein J.A., Thiolet C., Farret O., Buisson Y., Molinie C. (2000). Subfulminant hepatitis E in France. Gastroenterol. Clin. Biol..

[151] Skidmore S.J., Yarbough P.O., Gabor K.A., Tam A.W., Reyes G.R., Flower A.J. (1991). Imported Hepatitis E in UK. Lancet.

[152] Abid M., O’Brien S.J., Boxall E.H., Skidmore S.J. (1997). Hepatitis and travel abroad: A case report. J. Travel. Med..

[153] Zaaijer H.L., Kok M., Lelie P.N., Timmerman R.J., Chau K., van der Pal H.J. (1993). Hepatitis E in The Netherlands: Imported and endemic. Lancet.

[154] Johansson P.J., Mushahwar I.K., Norkrans G., Weiland O., Nordenfelt E. (1995). Hepatitis E virus infections in patients with acute hepatitis non-A-D in Sweden. Scand. J. Infect Dis..

[155] Ijaz S., Arnold E., Banks M., Bendall R.P., Cramp M.E., Cunningham R., Dalton H.R., Harrison T.J., Hill S.F., Macfarlane L. (2005). Non-travel-associated hepatitis E in England and Wales: Demographic, clinical, and molecular epidemiological characteristics. J. Infect. Dis..

[156] Riezebos-Brilman A., Verschuuren E.A., van Son W.J., van Imhoff G.W., Brügemann J., Blokzijl H., Niesters H.G. (2013). The clinical course of hepatitis E virus infection in patients of a tertiary Dutch hospital over a 5-year period. J. Clin. Virol..

[157] Dalton H.R., Thurairajah P.H., Fellows H.J., Hussaini H.S., Mitchell J., Bendall R., Banks M., Ijaz S., Teo C.G., Levine D.F. (2007). Autochthonous hepatitis E in southwest England. J. Viral. Hepat..

[158] Preiss J.C., Plentz A., Engelmann E., Schneider T., Jilg W., Zeitz M., Duchmann R. (2006). Autochthonous hepatitis E virus infection in Germany with sequence similar other European isolates. Infection.

[159] Brost S., Wenzel J.J., Ganten T.M., Filser M., Flechtenmacher C., Boehm S., Astani A., Jilg W., Zeier M., Schnitzler P. (2010). Sporadic cases of acute autochthonous hepatitis E virus infection in Southwest Germany. J. Clin. Virol..

[160] Krüttgen A., Scheithauer S., Häusler M., Kleines M. (2011). First report of an autochthonous hepatitis E virus genotype 3 infection in a 5 month old female child in Germany. J. Clin. Virol..

[161] Gauss A., Wenzel J.J., Flechtenmacher C., Navid M.H., Eisenbach C., Jilg W., Stremmel W., Schnitzler P. (2012). Chronic hepatitis E virus infection in a patient with leukemia and elevated transaminases: A case report. J. Med. Case Rep..

[162] Tabatabai J., Wenzel J.J., Soboletzki M., Flux C., Navid M.H., Schnitzler P. (2014). First case report of an acute hepatitis E subgenotype 3c infection during pregnancy in Germany. J. Clin. Virol..

[163] Vollmer T., Diekmann J., Johne R., Eberhardt M., Knabbe C., Dreier J. (2012). Novel approach for detection of hepatitis E virus infection in German blood donors. J. Clin. Microbiol..

[164] Vasickova P., Slany M., Chalupa P., Holub M., Svoboda R., Pavlik I. (2011). Detection and phylogenetic characterization of human hepatitis E virus strains, Czech Republic. Emerg. Infect. Dis..

[165] Chalupa P., Vasickova P., Pavlik I., Holub M. (2014). Endemic hepatitis E in the Czech Republic. Clin. Infect. Dis..

[166] Colson P., Borentain P., Motte A., Lagrange X., Kaba M., Henry M., Tamalet C., Gérolami R. (2007). First human cases of hepatitis E infection with genotype 3c strains. J. Clin. Virol..

[167] Doudier B., Vencatassin H., Aherfi S., Colson P. (2014). Fatal fulminant hepatitis Eassociated with autoimmune hepatitis and excessive paracetamol intake in Southeastern France. J. Clin. Microbiol..

[168] Renou C., Cadranel J.F., Bourlière M., Halfon P., Ouzan D., Rifflet H., Carenco P., Harafa A., Bertrand J.J., Boutrouille A. (2007). Possible zoonotic transmission of hepatitis E from pet pig to its owner. Emerg. Infect. Dis..

[169] Bouquet J., Tessé S., Lunazzi A., Eloit M., Rose N., Nicand E., Pavio N. (2011). Close similarity between sequences of hepatitis E virus recovered from humans and swine, France, 2008–2009. Emerg. Infect. Dis..

[170] Legrand-Abravanel F., Kamar N., Sandres-Saune K., Garrouste C., Dubois M., Mansuy J.M., Muscari F., Sallusto F., Rostaing L., Izopet J. (2010). Characteristics of autochthonous hepatitis E virus infection in solid-organ transplant recipients in France. J. Infect. Dis..

[171] Moal V., Gerolami R., Colson P. (2012). First human case of co-infection with two different subtypes of hepatitis E virus. Intervirology.

[172] Doudier B., Verrot D., Serratrice C., Poucel C., Auguste R., Colson P. (2015). Fatal outcome of autochthonous hepatitis E in a patient with B cell lymphoma in Southeastern France. J. Clin. Microbiol..

[173] Colson P., Borentain P., Queyriaux B., Kaba M., Moal V., Gallian P., Heyries L., Raoult D., Gerolami R. (2010). Pig liver sausage as a source of hepatitis E virus transmission to humans. J. Infect. Dis..

[174] Riezebos-Brilman A., Puchhammer-Stöckl E., van der Weide H.Y., Haagsma E.B., Jaksch P., Bejvl I., Niesters H.G., Verschuuren E.A. (2013). Chronic hepatitis E infection in lung transplant recipients. J. Heart Lung Transplant..

[175] Fogeda M., Avellón A., Cilla C.G., Echevarría J.M. (2009). Imported and autochthonous hepatitis E virus strains in Spain. J. Med. Virol..

[176] Anty R., Ollier L., Péron J.M., Nicand E., Cannavo I., Bongain A., Giordanengo V., Tran A. (2012). First case report of an acute genotype 3 hepatitis E infected pregnant woman living in South-Eastern France. J. Clin. Virol..

[177] Mateos Lindemann M.L., Morales J.G., Fernández-Barredo S., Domínguez M.R., García de la Hoz F., Halfon P., Pérez Gracia M.T. (2010). Fulminant hepatitis E in a woman taking oral contraceptive medication. Am. J. Trop. Med. Hyg..

[178] Mateos-Lindemann M.L., Diez-Aguilar M., González-Galdamez A., Graus-Morales J., Moreno-Zamora A., Perez-Gracia M.T. (2013). Acute, chronic and fulminant hepatitis E: Seven years of experience (2004–2011). Enferm. Infec. Microbiol. Clin..

[179] Idolo A., Serio F., Lugoli F., Grassi T., Bagordo F., Guido M., Privitera G., Lobreglio G., de Donno A. (2013). Identification of HEV in symptom-free migrants andenvironmental samples in Italy. J. Viral Hepat..

[180] Garbuglia A.R., Alessandrini A.I., Pavio N., Tessé S., Grignolo S., Viscoli C., Lapa D., Capobianchi M.R. (2015). Male patient with acute hepatitis E in Genoa, Italy: Figatelli (pork liver sausage) as probable source of the infection. Clin. Microbiol. Infect..

[181] Pfefferle S., Frickmann H., Gabriel M., Schmitz N., Günther S., Schmidt-Chanasit J. (2012). Fatal course of an autochthonous hepatitis E virus infection in a patient with leukemia in Germany. Infection.

[182] Goyal R., Kumar A., Panda S.K., Paul S.B., Acharya S.K. (2012). Ribavirin therapy for hepatitis E virus-induced acute on chronic liver failure: A preliminary report. Antivir. Ther..

[183] Pischke S., Hardtke S., Bode U., Birkner S., Chatzikyrkou C., Kauffmann W., Bara C.L., Gottlieb J., Wenzel J., Manns M.P. (2013). Ribavirin treatment of acute and chronic hepatitis E: A single-centre experience. Liver Int..

[184] Gerolami R., Borentain P., Raissouni F., Motte A., Solas C., Colson P. (2011). Treatment of severe acute hepatitis E by ribavirin. J. Clin. Virol..

[185] Hegenbarth K., Maurer U., Kroisel P.M., Fickert P., Trauner M., Stauber R.E. (2001). No evidence for mutagenic effects of ribavirin: Report of two normal pregnancies. Am. J. Gastroenterol..

[186] Rezvani M., Koren G. (2006). Pregnancy outcome after exposure to injectable ribavirin during embryogenesis. Reprod. Toxicol..

[187] Pischke S., Stiefel P., Franz B., Bremer B., Suneetha P.V., Heim A., Ganzenmueller T., Schlue J., Horn-Wichmann R., Raupach R. (2012). Chronic hepatitis e in heart transplant recipients. Am. J. Transplant..

[188] Te H.S., Drobeniuc J., Kamili S., Dong C., Hart J., Sharapov U.M. (2013). Hepatitis E virus infection in a liver transplant recipient in the United States: A case report. Transplant. Proc..

[189] Kamar N., Rostaing L., Abravanel F., Garrouste C., Esposito L., Cardeau-Desangles I., Mansuy J.M., Selves J., Peron J.M., Otal P. (2010). Pegylated interferon-alpha for treating chronic hepatitis E virus infection after liver transplantation. Clin. Infect. Dis..

[190] Haagsma E.B., Riezebos-Brilman A., van den Berg A.P., Porte R.J., Niesters H.G. (2010). Treatment of chronic hepatitis E in liver transplant recipients with pegylated interferon α-2b. Liver Transplant..

[191] Neukam K., Barreiro P., Macías J., Avellón A., Cifuentes C., Martín-Carbonero L., Echevarría J.M., Vargas J., Soriano V., Pineda J.A. (2013). Chronic hepatitis E in HIV patients: Rapid progression to cirrhosis and response to oral ribavirin. Clin. Infect. Dis..

[192] Hajji H., Gérolami R., Solas C., Moreau J., Colson P. (2013). Chronic hepatitis Eresolution in a human immunodeficiency virus (HIV)-infected patient treated with ribavirin. Int. J. Antimicrob. Agents.

[193] Jagjit Singh G.K., Ijaz S., Rockwood N., Farnworth S.P., Devitt E., Atkins M., Tedder R., Nelson M. (2013). Chronic Hepatitis E as a cause for cryptogenic cirrhosis in HIV. J. Infect..

[194] Dalton H.R., Keane F.E., Bendall R., Mathew J., Ijaz S. (2011). Treatment of chronic hepatitis E in a patient with HIV infection. Ann. Intern. Med..

[195] Miyamura T. (2011). Hepatitis E virus infection in developed countries. Virus Res..

[196] Khudyakov Y., Kamili S. (2011). Serological diagnostics of hepatitis E virus infection. Virus Res..

[197] Pas S.D., Streefkerk R.H., Pronk M., de Man R.A., Beersma M.F., Osterhaus A.D., van der Eijk A.A. (2013). Diagnostic performance of selected commercial HEV IgM and IgG ELISAs for immunocompromised and immunocompetent patients. J. Clin. Virol..

[198] Drobeniuc J., Meng J., Reuter G., Greene-Montfort T., Khudyakova N., Dimitrova Z., Kamili S., Teo C.G. (2010). Serologic assays specific to immunoglobulin M antibodies against hepatitis E virus: Pangenotypic evaluation of performances. Clin. Infect. Dis..

[199] Bendall R., Ellis V., Ijaz S., Ali R., Dalton H. (2010). A comparison of two commercially available anti HEV IgG kits and a re-evaluation of anti-HEV IgG seroprevalence data in developed countries. J. Med. Virol..

[200] Schnegg A., Bürgisser P., André C., Kenfak-Foguena A., Canellini G., Moradpour D., Abravanel F., Izopet J., Cavassini M., Darling K.E. (2013). An analysis of the benefit of using HEV genotype 3 antigens in detecting anti-HEV IgG in a European population. PLoS ONE.

[201] Avellon A., Morago L., Garcia-Galera M.D., Munoz M., Echevarría J.M. (2015). Comparative sensitivity of commercial tests for hepatitis E genotype 3 virus antibody detection. J. Med. Virol..

[202] Di Bartolo I., Angeloni G., Ponterio E., Ostanello F., Ruggeri F.M. (2015). Detection of hepatitis E virus in pork liver sausages. Int. J. Food Microbiol..

[203] Renou C., Roque-Afonso A.M., Pavio N. (2014). Foodborne transmission of hepatitis E virus from raw pork liver sausage, France. Emerg. Infect. Dis..

[204] Serracca L., Battistini R., Rossini I., Mignone W., Peletto S., Boin C., Pistone G., Ercolini R., Ercolini C. (2015). Molecular investigation on the presence of hepatitis E virus (HEV) in wild game in north-western Italy. Food Environ. Virol..

[205] Martin-Latil S., Hennechart-Collette C., Guillier L., Perelle S. (2014). Method for HEV detection in raw pig liver products and its implementation for naturally contaminated food. Int. J. Food Microbiol..

[206] Boxall E., Herborn A., Kochethu G., Pratt G., Adams D., Ijaz S., Teo C.G. (2006). Transfusion-transmitted hepatitis E in a “non hyperendemic” country. Transfus. Med..

[207] Colson P., Coze C., Gallian P., Henry M., de Micco P., Tamalet C. (2007). Transfusion-associated hepatitis E, France. Emerg. Infect. Dis..

